# Harnessing the potential of machine learning and artificial intelligence for dementia research

**DOI:** 10.1186/s40708-022-00183-3

**Published:** 2023-02-24

**Authors:** Janice M. Ranson, Magda Bucholc, Donald Lyall, Danielle Newby, Laura Winchester, Neil P. Oxtoby, Michele Veldsman, Timothy Rittman, Sarah Marzi, Nathan Skene, Ahmad Al Khleifat, Isabelle F. Foote, Vasiliki Orgeta, Andrey Kormilitzin, Ilianna Lourida, David J. Llewellyn

**Affiliations:** 1 University of Exeter Medical School, College House, St Luke’s Campus, Heavitree Road, Exeter, EX1 2LU UK; 2 Cognitive Analytics Research Lab, School of Computing, Engineering & Intelligent Systems, Ulster University, Derry, UK; 3 Institute of Health and Wellbeing, University of Glasgow, Glasgow, UK; 4 Department of Psychiatry, University of Oxford, Oxford, UK; 5 Department of Computer Science, UCL Centre for Medical Image Computing, University College London, London, UK; 6 Cambridge Cognition, Cambridge, UK; 7 Department of Clinical Neurosciences, University of Cambridge, Cambridge, UK; 8 UK Dementia Research Institute, Imperial College London, London, UK; 9 Department of Brain Sciences, Imperial College London, London, UK; 10 Department of Basic and Clinical Neuroscience, King’s College London, London, UK; 11 University of Colorado Boulder, Boulder, USA; 12 Division of Psychiatry, University College London, London, UK; 13 The Alan Turing Institute, London, UK

**Keywords:** Dementia, Artificial intelligence, Machine learning, Genetics, Drug discovery, Neuroimaging, Prevention, iPSC, Animal models

## Abstract

Progress in dementia research has been limited, with substantial gaps in our knowledge of targets for prevention, mechanisms for disease progression, and disease-modifying treatments. The growing availability of multimodal data sets opens possibilities for the application of machine learning and artificial intelligence (AI) to help answer key questions in the field. We provide an overview of the state of the science, highlighting current challenges and opportunities for utilisation of AI approaches to move the field forward in the areas of genetics, experimental medicine, drug discovery and trials optimisation, imaging, and prevention. Machine learning methods can enhance results of genetic studies, help determine biological effects and facilitate the identification of drug targets based on genetic and transcriptomic information. The use of unsupervised learning for understanding disease mechanisms for drug discovery is promising, while analysis of multimodal data sets to characterise and quantify disease severity and subtype are also beginning to contribute to optimisation of clinical trial recruitment. Data-driven experimental medicine is needed to analyse data across modalities and develop novel algorithms to translate insights from animal models to human disease biology. AI methods in neuroimaging outperform traditional approaches for diagnostic classification, and although challenges around validation and translation remain, there is optimism for their meaningful integration to clinical practice in the near future. AI-based models can also clarify our understanding of the causality and commonality of dementia risk factors, informing and improving risk prediction models along with the development of preventative interventions. The complexity and heterogeneity of dementia requires an alternative approach beyond traditional design and analytical approaches. Although not yet widely used in dementia research, machine learning and AI have the potential to unlock current challenges and advance precision dementia medicine.

## Introduction

**Fig. 1:**
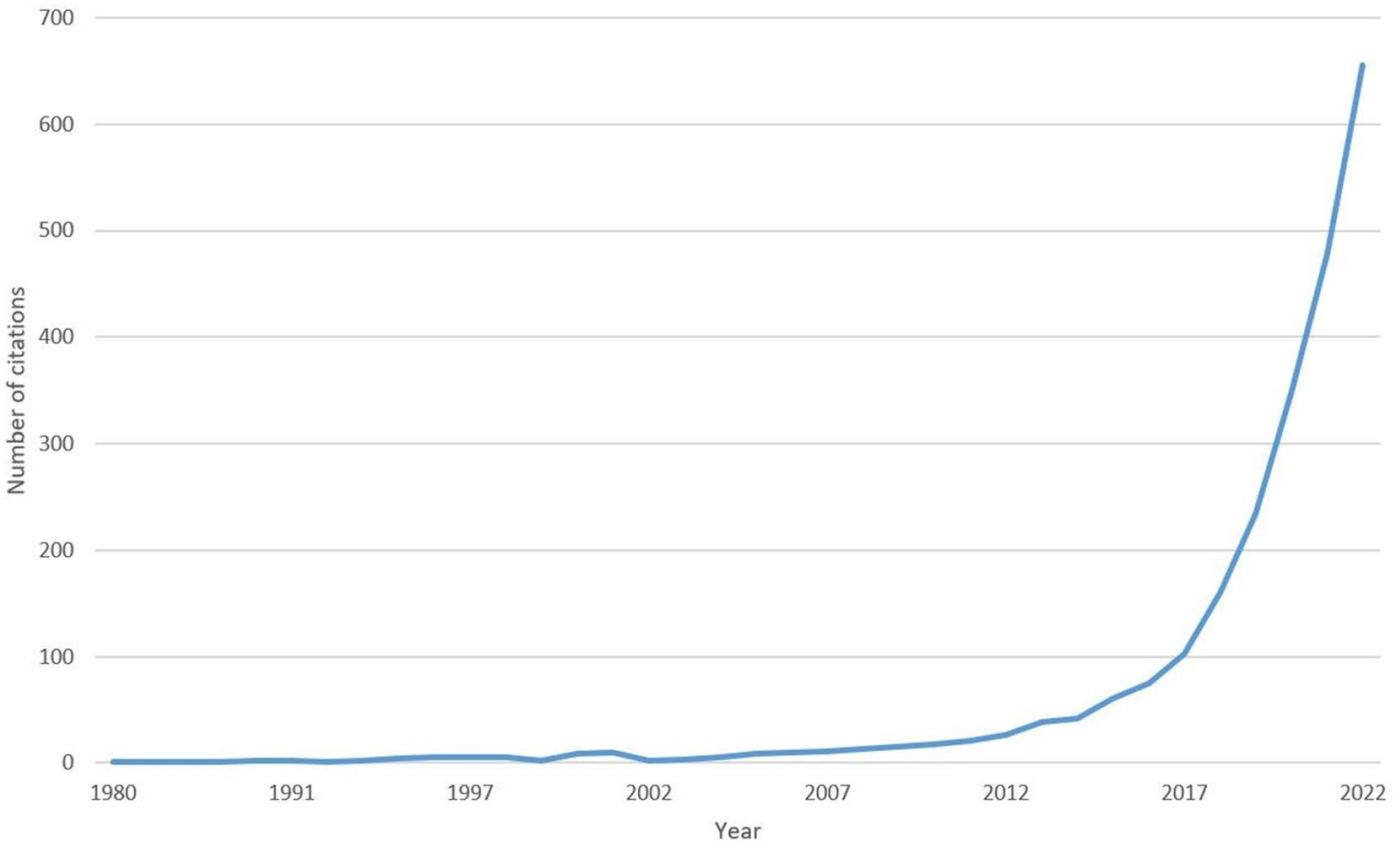
Growth in citations related to AI in dementia research. Source: PubMed citations using the search term (Alzheimer*[Title/Abstract] OR dement*[Title/Abstract]) AND (AI[Title/Abstract] OR artificial intelligence[Title/Abstract] OR machine learning[Title/Abstract])

Dementia is a syndrome caused by an acquired and sustained decline in brain function, leading to difficulty with everyday activities. With multiple underlying aetiologies, clinical presentation can vary and includes problems with memory, attention, reasoning and judgement, communication, language, and even visual perception [1]. Many people living with dementia remain undiagnosed, and clinicians struggle to deal with this complex and variable condition for which there is currently no cure. The intimidating challenge that dementia represents reflects the sheer complexity of the human brain. Dementia research has been historically neglected, reflecting the belief that dementia is part of normal ageing or that it’s simply too difficult to solve. The realisation that adults age differently, and that the presence of disease does not necessarily lead to the clinical syndrome of dementia, has led to a more optimistic outlook. Research in dementia aims to identify risk factors, potential mechanisms for disease progression, and development of treatments for symptom alleviation and disease modification. However, progress has been limited and we are still looking for better ways to prevent, predict and treat this condition. There is an increasing availability of rich, multimodal data from a variety of sources, including pre-clinical experimental data, genetic, imaging and phenotypic information from population-based cohorts and clinical trials, clinical data from electronic health records, and real-world measurements from digital wearables. Therefore, the conventional approach of specialists working in silos, generating relatively small-scale data analysed using traditional statistics, no longer seems suited to combat dementia. There is growing interest in analysing and combining data in novel and innovative ways, and AI and machine learning offer an alternative approach which can help to embrace the complexity and heterogeneity which characterises dementia. As shown in Fig. 1, the field of AI applied to dementia research is relatively new though rapidly growing. By the end of 2022, there were 2385 articles with the majority published in 2020 or later.

Here, we provide an overview of the state of the science in 5 key areas, where AI has the potential to achieve impact and enhance dementia research and healthcare: genetics and omics, experimental medicine, drug discovery and trials optimisation, imaging, and prevention.

## Genetics

Now is an exceptional time to be a data scientist working with genomic data to gain insight into the aetiology of dementia. Data-generating methods are proliferating rapidly, spurred on by the falling costs of DNA sequencing and microfluidic technologies which have enabled single cell genomics. A major advance enabling the application of data science to dementia is the emergence of reasonably well-powered genome-wide association studies (GWAS): there are now 38 genome-wide significant risk loci which have been discovered from studies of a total 1,126,563 participants [2] While 38 loci does not sound as though it falls within the realm of ‘big data’, recently developed statistical methods such as linkage disequilibrium score regression [3] and MAGMA [4] have enabled the full genomic signal to be considered by accounting for confound from linkage disequilibrium (i.e., correlation of nearby variants). Many hurdles stand between us and a clear understanding of dementia aetiology though [5]: we do not know what effect most genetic variants have; we do not know how they relate to cellular changes seen in disease; nor how genetic factors interact with modifiable risks. Integrative data set analyses are likely to help answer these questions.

### How do we determine the biological effect of genetic variants?

The results of a GWAS tell us which variants are associated with disease. While these are commonly discussed as Single Nucleotide Polymorphisms (SNPs), it should not be assumed that this means we know the actual base pair in the genome responsible for increased disease risk. Alleles found at nearby genetic variants are correlated, known as linkage disequilibrium. A family of statistical techniques have been developed to determine which genetic variant in a region is likely to be causal, by considering conditional probabilities and functional (e.g., epigenetic) annotations [6]. Supervised learning is a useful adjunct to statistical fine mapping: the genomic features associated with known expression-quantitative trait loci were used to train a classifier, whose output from new variants could then be used to set the priors for statistical fine mapping [7]. Identifying the causal variant does not tell us what those variants do though; toward this end, various machine learning models [8] has been developed to predict genomic features, such as transcription factor binding [9], Ribonucleic Acid (RNA) protein binding [10], RNA splicing [11], and 3D genome structure [12]. These models use cutting-edge machine learning techniques, such as attentional networks, to enable predictions of variant effects to be made over long distances (e.g., 100,000 bp) [9].

### How do we get from genetic epidemiology and -omics to practical applications?

GWAS investigate the associations of dementia with genetic variants across the genome, and individual associations often have relatively small effect sizes.

Early stage clinical drug trials that are successful have substantial corroboration in independent GWAS, and targets with genetic evidence of disease association are almost twice as likely to succeed across multiple phases [13]. Similarly, GWAS provides an opportunity to identify existing drugs which could be repurposed from their original target condition on the basis of shared targets [14]. This suggests that clinical drug trials could be substantially more efficient in terms of duration and cost, on the basis of genetic and transcriptomic information. Polygenic risk scores are single values which reflect an individual’s cumulative, additive genetic risk for a trait, and which are often applied as predictors of traits in independent cohorts. Common complex traits and disorders primarily have polygenic, high-frequency but low-penetrance architectures. These scores have substantial potential utility in predicting lifetime risk of dementia. However, major challenges remain including how these scores can be used reliably at the individual level, their application across ethnicities, and whether their applicability varies at different stages of the lifecourse [15], plus the potential use of genetics and wide-ranging -omics generally in predictive modelling and machine learning [16, 17].

Genetic information can be leveraged, whereby variants in a gene that encode for a drug target are used to predict the on- and off-target effects of pharmacological modification. One example is the use of PCSK9 SNPs to mimic the low-density lipoprotein cholesterol-lowering PCSK9 inhibitor drug, where genetic evidence showed an additional association with increased risk of type-2 diabetes [18]. There is substantial scope to expand this approach to neurodegenerative and dementia-related outcomes, especially where expensive randomized controlled trials are underpowered to test for rare or particularly long-term outcomes including accelerated cognitive decline.

### How can we best communicate genetic/-omic information, and what it means, to real people?

There is evidence that members of the general public want to know their genetic risk for health conditions [19]. However, clinical dementia services do not consistently provide genotyping or testing for purported common dementia risk polymorphisms (e.g., Apolipoprotein E [APOE]). It is possible that the genetic risk of developing dementia may be mitigated by adherence to a healthy lifestyle. However, there is generally poor understanding regarding the interplay of modifiable risk factors, such as smoking, problem drinking and/or sedentary behaviour, and ‘risk’ genotypes [20]. For example, studies that examined whether the risk reduction associated with healthy lifestyle varies by APOE ε4 genotype have been inconsistent: some findings have shown beneficial effects of a multidomain lifestyle intervention irrespective of APOE ε4 status [21], whereas others indicate that following a healthier lifestyle is associated with reduced dementia risk only in APOE ε4 non-carriers [22]. Addressing both groups of factors in combinations such as in the form of composite lifestyle scores and polygenic risk scores for Alzheimer’s disease and dementia risk has also shown mixed evidence. A recent large observational study [23] suggested that genetic and lifestyle factors are independent and additive; a favourable lifestyle profile was associated with reduced dementia risk across genetic groups including those with a high genetic risk. In contrast, another large cohort study [24] found that a healthy lifestyle could not offset high genetic risk, and the observed associations of a favourable lifestyle against dementia were most pronounced for younger individuals at low genetic risk. In short, while we can say that there are genetic and environmental risk factors for dementia, we cannot clearly tell people whether one increases vulnerability to the other. In terms of the multitudes of -omics, there is a significant challenge in determining added value from more detailed phenotyping, e.g., for telomere length or specific methylation sites, in terms of risk prediction [25].

Translating data into usable information which benefits public health is a major challenge. Areas in which AI and machine learning can help include meta-analyses of GWAS and ribonucleic acid sequencing data, development of polygenic scores for dementia subtypes, and combining -omics data to enhance our understanding of functional implications of identified genetic associations. A fundamental priority of genetics in dementia is to successfully integrate these promising approaches for real-world impact.

## Experimental medicine

Animal [24] and cellular [25] models can provide vital evidence of mechanism. These are used to dissect and better understand disease hypotheses and potential causal pathways. These results can give rise to new drug targets, and discovery of new biomarkers to allow identification of dementia-related diseases in their preclinical form. Experimental models for dementia include a spectrum of mice with specific genetic mutations or knock-ins [26], patient-derived induced pluripotent stem cell (iPSC) cultures, and human tissues [27, 28]. We have additionally seen the development of complex multi-cellular and multi-species models, including organoids of the human brain [28, 29], multi-species models of the blood–brain barrier [30] and chimeric mouse models, containing live human cells [31, 32]. All these models capture different aspects and states of disease biology and allow varying extents of control over genetic and environmental experimental intervention.

Current developments in intelligent experimental medicine encapsulate some of the most promising opportunities for innovation using AI in dementia research. Data-driven experimental medicine is essential to tackle the challenge of integrative analyses across multiple studies and heterogeneous model systems. Advanced informatics methodologies can detect results that might be missed by a direct analysis. For example, by integrating data modalities within a single cohort researchers can make stronger inferences of underlying changes and specific patterns. Using machine learning and AI, researchers can find new associations or interactions that may be obscured by data noise or restrictive traditional methods to make new disease inferences.

Integrating across biological modalities and incorporating aspects of AI-computation, simulated digital brains, which recapture brain circuitry and biological processes at different scales (e.g., microcircuitry involving individual synapses, or global brain activation patterns) are a new class of model that holds great promise for basic neuroscience research and personalized medicine applications [33]. In particular, the development of digital twin brain models, which link patient specific biological features, including brain structure, pathology and functional characteristics with an in silico brain, could revolutionize drug development and individualized treatment and rehabilitation in neurological conditions.^34^

To progress the field of experimental medicine in dementia research, three key questions need to be addressed.

### What makes a good experimental model?

To quantify validity in modern experimental medicine, we need measurable criteria for how to determine suitability and representativeness of experimental models. Which aspects of disease biology can be captured by various model systems? For example, given that iPSC-derived cell cultures or organoids show gene regulatory ageing markers of very early development, do they represent suitable models of neurodegenerative disease processes in the context of old age? Or could ageing signatures even be simulated experimentally or computationally in these in vitro systems?

### How can we make best use of multimodal data?

Robust studies should control for experimental factors and batch effects that can have an impact on the measured phenotype, by harmonising, randomising across experimental conditions and including appropriate covariates in analyses. This is challenging for small, single modality studies, with limited statistical power and reliability. Development of analytic methods and tools that can span across modalities and leverage these links, is a rapidly evolving and promising field. Connecting brain activity with gene expression patterns, for example, could give new insights into gene regulation in the context of functional activation of neurons. In the same way, atlases across phenotypes and omics, carefully collected on matched samples are set to provide novel insights into disease biology [35, 36].

### How can we translate insights from experimental models to human disease biology?

Clinical trials for drugs developed using animal models are generally unsuccessful [37, 38]. We need strong quantitative approaches for cross-model translation. Machine learning approaches can be leveraged to translate gene-regulatory networks and the response to experimental perturbation across species. By reviewing existing approaches and those used in other fields, we can address these kinds of translational challenges. Armed with prior biological knowledge and large-scale reference data sets for baseline and perturbed conditions, development of novel translation algorithms is possible.

## Drug discovery and trials optimisation

Despite a concerted and sustained international research effort in developing and testing disease-modifying therapies for dementia, progress has been poor. For Alzheimer’s disease, there are a small number of treatments which can produce short-term symptomatic relief, but the disease continues unabated. A plethora of experimental drugs have been tested in hundreds of clinical trials over the past two decades. However, so far only Aducanumab has been approved by the US Food and Drug Administration [39–41]. Data science, AI and related methods will prove invaluable for addressing these challenges, by improving the precision of predictions and making the most of available data [42, 43]. There is growing interest in applying data science methods to enhance clinical trials and computational drug discovery, such as using unsupervised learning to discover unseen patterns in data [44], and meta models for understanding disease mechanisms [45, 46].

Clinical trials aim to recruit at-risk individuals who may benefit from an experimental drug. The ideal scenario is to recruit a large, population-representative sample of individuals at the same stage of disease progression or with the same risk of developing a disease. In reality, sample sizes are limited by financial budgets, and assessment of disease severity and risk is not straightforward; these diseases are heterogeneous and lack a well-defined disease progression axis [47–49]. In an attempt to overcome this, recent efforts to improve recruitment and screening for clinical trials in Alzheimer’s disease have included use of biomarker data [50–52]. This reflects a shift in the research definition of Alzheimer’s disease to incorporate biomarkers, especially in the pre-symptomatic phase [53–56]. Current biomarker-based screening is quite crude; for example, values are typically dichotomised using predefined cut points which may have contributed to the failure of biomarker-based screening to improve the low success rate for clinical trials in Alzheimer’s disease [48, 57, 58]. There is much room for improvement.

Identifying of suitable candidates for clinical trials is a challenge well-suited to advanced data science methods. Multimodal data from large cohort and population studies can be used [42, 43] to characterise and quantify disease severity [59, 60] and subtype [61] even in the absence of a well-defined disease progression axis [62]. Such approaches promise the precision that has been hampering clinical trials to date [63].

While data from large observational studies is becoming increasingly available, this is not the case for interventional studies. Patient-level data from clinical trials is needed to perform some of the analyses we envisage [64, 65]. However, the pharmaceutical sponsors of clinical trials in neurology have a track record of being extremely protective of this data. Indeed, most pharma companies involved in Alzheimer’s disease clinical trials have an explicit policy only to share patient-level data after regulatory approval of an experimental drug. Opportunities for optimising clinical trials include utilising publicly available data, and improving the state of the art in precision understanding and forecasting of dementias [16, 66]. The former lends itself to traditional data science methods (e.g., gradient boosting for feature selection or weighting) and AI (e.g., feature generation from neuroimaging data). The latter is an active area of research that will benefit from large multidisciplinary collaborative initiatives leveraging available data. Examples include data science competitions such as the TADPOLE Challenge [67] and international collaborations focussed on novel statistical methods such as the series of workshops led by the EuroPOND consortium (http://europond.eu) or the Fraunhofer SCAI (http://www.diseaseprogressionmodels.eu/).

Another challenge is that following participants over the time which dementia typically develops is generally impractical and prohibitively expensive, particularly for dementia prevention trials. One low-cost solution is interrogation of electronic health records to identify patients who are subsequently diagnosed with dementia or experience adverse events. Relevant information is often recorded in free-text clinical notes which can be problematic for conventional analytic approaches [68]. Advances in text mining and natural language processing facilitate analysis of relevant information. Using population-level electronic health record data, this approach can also inform future care by identifying how new treatments benefit patient subgroups, and develop drug-response predictive models, thus capturing real-world effectiveness.

## Neuroimaging

Structural neuroimaging using Computed Tomography (CT) or Magnetic Resonance Imaging (MRI) is routine in the diagnosis and management of dementia, providing insight into differential diagnosis and excluding other causes of cognitive impairment. Additional neuroimaging modalities can help analyse: (1) brain activity (often referred to as functional neuroimaging) using functional MRI [69], electroencephalography [70] or magnetoencephalography [71, 72]; (2) metabolic changes using positron emission tomography (PET); (3) specific pathologies using PET ligands for protein aggregation, such as beta-amyloid [73, 74] or tau [75].

Neuroimaging generates large, complex data which is increasingly beyond the ability of human interpretation or traditional statistical approaches, but ideally suited to AI methods for understanding disease mechanisms and supporting clinical diagnosis. Large data sets have accelerated the development of AI tools, including the Alzheimer's Disease Neuroimaging Initiative [76], National Alzheimer's Coordinating Center [77], Open Access Series of Imaging Studies [78], Genetic Frontotemporal dementia Initiative [79], and data repositories, such as Dementias Platform UK [80].

Over 250 AI studies of neuroimaging have addressed clinical questions of dementia diagnosis or prognosis. AI methods in structural MRI outperform traditional approaches when compared head-to-head, for example, using hippocampal volume for diagnostic classification [81, 82], or conversion from mild cognitive impairment to Alzheimer’s disease. One promising approach combined structural MRI, PET and clinical data to train a machine learning model that subsequently predicted conversion from mild cognitive impairment to Alzheimer’s disease using structural MRI alone [83, 84]. While barriers remain in validation and translation to the clinic, we are optimistic that meaningful AI decision support tools for neuroimaging will be in clinical practice within the next decade [85].

The anatomical specificity of neuroimaging enables more than simple classification, it can provide insights into disease mechanisms of neurodegeneration across the brain. The first study to apply a ‘big data’ approach to this challenge used multimodal MRI and PET imaging combined with plasma and cerebrospinal fluid markers in a multifactorial generative model to infer that vascular changes occurred earliest in Alzheimer’s disease [86]. This approach using neuroimaging, with or without other biomarkers, to infer disease progression has since been extended, most notably using the Subtype and Stage Interference model. This model has been applied to genetic frontotemporal dementia [61] investigating the complex relationship between genotype and pattern of structural brain changes, and more recently in Alzheimer’s disease to identify four distinct patterns of tau accumulation [87]. We expect AI applied to neuroimaging to continue to shed light on the interplay between mechanisms of neurodegeneration across the brain.

The issue of interpretability remains a challenge, particularly with the increasing use of ‘black box’ deep learning methods [88, 89]. Understanding the features used for classification is important to relate changes to anatomical brain regions, and to ensure that classification is not based on noise signals, such as motion in functional imaging methods. This can be achieved using simpler ‘white-box’ machine learning models, where model features are readily accessible. Various methods to identify features within neural networks are emerging and have been successfully applied to neuroimaging data in Alzheimer’s disease [90].

While we are optimistic about AI in neuroimaging for dementia, some challenges remain [91]. One key challenge is ensuring that research populations represent the real-world setting they are drawn from. AI is notoriously sensitive to bias within the data that trains its algorithms, and selection bias has been highlighted in data sets of neuroimaging for dementia. Future data collection must recruit strategically to address this issue.

A second key challenge is the practical issue of regulatory approval. Only four AI methods have been approved for clinical use in neuroimaging for dementia in the US or EU, but most of these perform image segmentation, and none directly classify patient groups [92]. This low rate of success suggests a significant barrier at the point of translation from research to clinical practice. Neuroimaging has a central part to play in data science and AI applied to dementia research. The development of AI tools and increasing availability of richly phenotyped neuroimaging data means that now is the time to address the remaining challenges in our field and harness these powerful tools for the benefit of patients.

## Prevention

Epidemiological evidence shows a decrease in age-specific dementia incidence in recent birth cohorts, demonstrating the potential for dementia prevention by targeting modifiable risk factors [93, 94]. The most recent Lancet Commission on Dementia Prevention, Intervention and Care identified 12 modifiable risk factors, which could prevent up to 40% of new dementia cases [93]. These are: low education (early life); hearing loss, traumatic brain injury, hypertension, high alcohol intake, and obesity (midlife); smoking, depression, social isolation, physical inactivity, air pollution and diabetes (late life). Several recent systematic reviews have highlighted additional risk factors [95–98] with highly suggestive evidence emerging for sleep disturbances, stroke, benzodiazepine use, gait speed, vitamin D deficiency, and high homocysteine levels. Additional risk factors have been emphasised albeit the strength of evidence tends to fluctuate depending on the methodological criteria of the systematic review; these include cognitive inactivity, poor diet, hyperlipidaemia, atrial fibrillation, inflammatory markers, and anxiety [95–97]. Weaker evidence has been suggested for other frequently studied factors, such as renal dysfunction [95], coronary heart disease [95], tooth loss [96], and postoperative delirium [98]. The latest evidence indicates from observational studies indicates very weak or no significant association between cancer, general anaesthesia or non-steroidal anti-inflammatory drugs and dementia risk [98]. Among the above factors, education and plasma glucose levels are those with the strongest evidence supported by Mendelian randomisation causal analyses [98]. Finally, research focusing only on environmental exposures indicates there is some evidence for an association of air pollution, pesticides, aluminium, silicon, and electric and magnetic fields with increased dementia risk [99–101].

Current evidence highlights the multitude of risk factors and pathologies contributing to dementia risk. Machine learning and AI methods can be used to improve our understanding of commonality and causality of risk factors to develop and test effective preventative interventions.

A major challenge in this field is the issue of reverse causation. Many traditional statistical approaches fail to distinguish causal risk factors from prodromal dementia symptoms. Methods such as Mendelian randomization are providing new insights into causal pathways for risk factors [102], but can be inadequately powered and suffer from weak instrument and survival bias. Machine learning methods, such as deep learning approaches, could improve the way risk variants are identified and functionally assessed within genome-wide association studies [103–105], creating stronger genetic instruments to improve causal analysis. Contemporary causal machine learning approaches have the potential to enhance our understanding of the underlying mechanisms which connect multiple risk factors, pathologies and the clinical syndrome of dementia itself. An exhaustive search for causal structures using machine learning with high-dimensional health data is computationally unfeasible, although heuristic approaches to identify causal structures have been developed, such as fast causal inference [106].

Most dementia risk factors are moderately correlated [107, 108]. Despite this, many studies fail to consider the interactions between modifiable and non-modifiable risk factors (e.g., age and genetics) or non-linear effects. This leads to overly simplistic methods that do not reflect true biological relationships. For example, mid-life hypertension increases risk for dementia, but becomes apparently ‘protective’ during prodromal dementia [109–112], suggesting the onset of dementia itself can reduce blood pressure. Machine learning approaches can measure these interactions and nonlinear effects, which could uncover pathways that underpin the influence that multimorbidity has on dementia risk. Approaches, such as path signature-based methods [113], would allow us to model these complex relationships and identify the optimal duration and timing of lifestyle or drug interventions to reduce dementia risk.

Existing dementia risk prediction tools, such as the Cardiovascular Risk Factors, Aging, and Incidence of Dementia risk score, incorporate risk factors, such as age, education, physical activity, vascular and cardiometabolic risk factors, to predict dementia ~ 20 years later [114, 115]. However, these are based on linear models, leaving substantial scope for more advanced modelling, including extreme gradient boosting trees or deep neural networks.

Deep learning [116] and network-based approaches [117, 118], are being used to identify and validate existing drugs as potential preventative interventions to reduce risk and delay dementia onset [119]. Machine learning approaches can help clarify how and why certain drugs reduce risk, improving our understanding of dementia aetiology and promoting identification of new drug targets [120, 121]. The development of preventative interventions could in turn enable the use of personalised medicine by applying network meta-analytics [122] and other machine learning methods to recommend interventions and predict treatment response to improve patient outcomes [123].

Underpinning all these advances is the need for more high-quality, longitudinal and multimodal data, which currently remains unavailable from a single source. Better harmonisation of existing cohorts and multimodal data sets using AI methods will also help to inform guidance set out by policymakers and professional bodies for public health management.

## Multidisciplinary global collaborations for AI applied to dementia research and healthcare

The Deep Dementia Phenotyping (DEMON) Network is the first and largest global collaborative initiative which aims to transform dementia research and healthcare through the application of data science and AI (see www.demondementia.com). Launched in November 2019, the DEMON Network brings together over 1,500 members across six continents including dementia researchers, computer scientists, clinicians, AI specialists, third sector and industry representatives. Collaborative research, publications and knowledge transfer activities are conducted through practical Working Groups and Special Interest Groups covering all areas of dementia research. By coordinating and combining expertise and resources, the DEMON Network is leading a change in the way dementia research is conducted. The DEMON Network is largely resourced by academics volunteering their time. As the majority of research applying AI to dementia have so far addressed predictive and diagnostic challenges, this network is currently focused on dementia prevention and diagnosis, rather than dementia care.

In June 2022, a related Precision Dementia Medicine Interest Group was launched within the Alan Turing Institute, the UK national institute for data science and AI (https://www.turing.ac.uk/research/interest-groups/precision-dementia-medicine). In August 2022 a new Professional Interest Area in AI for Precision Dementia Medicine was launched within the Alzheimer’s Association International Society to Advance Alzheimer's Research and Treatment (ISTAART), which convenes the global Alzheimer's and dementia science community (https://action.alz.org/PersonifyEbusiness/Default.aspx?TabID=1753). Together, these initiatives aim to support the broad dementia research community to collaborate internationally, and share the common goal of enhancing dementia research using data science and AI.

## Conclusions

The quality of available data, power of analytic techniques, and infrastructure for collaboration are all improving rapidly. Table 1 summarises the current applications, challenges and prospects in each area of dementia research. The potential of the relatively new field of AI for dementia research remains largely unfulfilled, although some progress has been made and there is genuine cause for optimism (Box 1). Progress toward combating dementia and promoting brain health will be made more rapidly by bringing together the right data, the right analyses, and the right people. As a result, we anticipate that the development of AI and machine learning techniques will play a vital role in accelerating the pace of our discoveries.

**Table 1:** Overview of current applications, challenges and prospects for machine learning and AI applications in five key areas of dementia research

	Current areas of machine learning and AI applications	Challenges and knowledge gaps	Prospects and future directions
Genetics	Full genomic signal analysis [3, 4] Statistical fine mapping [6, 7] Single cell genomics [9, 10] Identification of causal variants [6]	Effect of specific genetic variants [5] Relation of genetic variation to cellular changes [5] Mixed evidence for interaction of genetics with modifiable risk [21−24]	Utilisation of integrative data sets [124] Combining omics data to identify functional implications [125] Application of genetic risk to individuals [15]
Experimental Medicine	Data-driven multimodal analysis [35, 36] Gene regulation [26, 27] Digital twin brain models link [33]structure, function and pathology	Translational gap from models to human disease biology [126] Lack of power in small, single modality studies [126] Poor reproducibility [127]	Efficient drug target discovery [128] Simulated ageing signatures [129] Digital brains for precision dementia research^34^
Drug discovery and Trials Optimisation	Intelligent drug target identification [16] Incorporation of multiple biomarker data [59] Natural language processing and text mining of electronic health records [68]	Heterogeneity of disease risk, severity and subtype [60, 61] Cost of longitudinal analysis [60] Restricted access to clinical trial data [64]	Enhanced identification of risk for trial recruitment [63] Utilising publicly available data and linked health records [16, 66] Multi institutional collaborative initiatives to share data [67]
Neuroimaging	Automated feature extraction for diagnosis and prediction [85] Combining imaging modalities and biomarker data [86] Investigation of disease progression and biological mechanisms [61, 87]	Lack of clinical implementation [85] Poor interpretability is challenging for regulation [91] Sensitivity to bias in the training data [91]	Validation of existing models for clinical settings [90] Availability of large data sets and repositories [85] Strategic recruitment to improve real-world applicability [91]
Prevention	Analysis of complex interactions in observational studies [113] Increased accuracy of polygenic risk and predictive models [15, 130] Validation of drug repurposing for dementia prevention [117, 118]	Inconsistent evidence for many potential risk factors [93, 95–97] Causal relationships poorly understood [98] Lack of statistical power [17]	Personalised dementia prevention interventions [122, 123] Deep learning for improved Mendelian randomisation [103, 105] Lifespan modelling to identify the optimal timing of a prevention intervention

## Box 1. Take-home points

**Table:** 

1) AI and machine learning techniques make it possible to analyse high-dimensional and multimodal data in a way that was not previously possible. 2) Current applications are most advanced in neuroimaging, where features can be efficiently and automatically extracted for incorporation into powerful diagnostic and predictive models. 3) New approaches in genetic discovery and dementia prevention include identification of interactions and causal variants, and development of more accurate polygenic risk scores. 4) Recent innovations in ‘intelligent’ experimental medicine are expected to facilitate more efficient drug target discovery, with powerful simulation studies and multimodal data approaches helping bridge the translational gap for new insights into human biology. 5) We now have an opportunity to improve clinical trials with more efficient targeting and recruitment strategies, with much optimism for a precision medicine approach to both primary and secondary prevention. 6) A coordinated multidisciplinary global approach is needed to bring the dementia research community together to achieve impact across these areas of promise.	

## Data Availability

Data sharing is not applicable to this review article as no new data were created or analysed in this study.

## Abbreviations

AI: Artificial intelligence
APOE: Apolipoprotein E
CT: Computed Tomography
DEMON: Deep Dementia Phenotyping Network
GWAS: Genome-Wide Association Studies
iPSC: Induced pluripotent stem cells
ISTAART: Alzheimer’s Association International Society to Advance Alzheimer's Research and Treatment
MRI: Magnetic Resonance Imaging
PET: Positron Emission Tomography
SNP: Single Nucleotide Polymorphism

## References

[1] Alzheimer’s Association. What Is Dementia? https://www.alz.org/alzheimers-dementia/what-is-dementia. Accessed 16 Feb 2019.

[2] Wightman DP, Jansen IE, Savage JE, et al. Largest GWAS (N=1,126,563) of Alzheimer’s Disease Implicates Microglia and Immune Cells. medRxiv 2020:2020.2011.2020.20235275.

[3] Finucane HK, Bulik-Sullivan B, Gusev A (2015). Partitioning heritability by functional annotation using genome-wide association summary statistics. Nat Genet.

[4] de Leeuw CA, Mooij JM, Heskes T, Posthuma D (2015). MAGMA: Generalized Gene-Set Analysis of GWAS Data. PLoS Comput Biol.

[5] Escott-Price V, Hardy J (2022). Genome-wide association studies for Alzheimer’s disease: bigger is not always better. Brain Commun.

[6] Weissbrod O, Hormozdiari F, Benner C (2020). Functionally informed fine-mapping and polygenic localization of complex trait heritability. Nat Genet.

[7] Wang QS, Kelley DR, Ulirsch J (2021). Leveraging supervised learning for functionally informed fine-mapping of cis-eQTLs identifies an additional 20,913 putative causal eQTLs. Nat Commun.

[8] Avsec Ž, Kreuzhuber R, Israeli J (2019). The Kipoi repository accelerates community exchange and reuse of predictive models for genomics. Nat Biotechnol.

[9] Avsec Ž, Agarwal V, Visentin D, et al. Effective gene expression prediction from sequence by integrating long-range interactions. bioRxiv 2021:2021.2004.2007.438649. 10.1038/s41592-021-01252-x PMC8490152 34608324

[10] Pan X, Shen H-B (2018). Predicting RNA–protein binding sites and motifs through combining local and global deep convolutional neural networks. Bioinformatics.

[11] Paggi JM, Bejerano G (2018). A sequence-based, deep learning model accurately predicts RNA splicing branchpoints. RNA.

[12] Schwessinger R, Gosden M, Downes D (2020). DeepC: predicting 3D genome folding using megabase-scale transfer learning. Nat Methods.

[13] King EA, Davis JW, Degner JF (2019). Are drug targets with genetic support twice as likely to be approved? Revised estimates of the impact of genetic support for drug mechanisms on the probability of drug approval. PLoS Genet.

[14] Visscher PM, Wray NR, Zhang Q (2017). 10 Years of GWAS discovery: biology, function, and translation. Am J Hum Genet.

[15] Lewis CM, Vassos E (2020). Polygenic risk scores: from research tools to clinical instruments. Genome Med.

[16] Myszczynska MA, Ojamies PN, Lacoste AMB (2020). Applications of machine learning to diagnosis and treatment of neurodegenerative diseases. Nat Rev Neurol.

[17] Rowe TW, Katzourou IK, Stevenson-Hoare JO, Bracher-Smith MR, Ivanov DK, Escott-Price V (2021). Machine learning for the life-time risk prediction of Alzheimer’s disease: a systematic review. Brain Communications.

[18] Schmidt AF, Swerdlow DI, Holmes MV (2017). PCSK9 genetic variants and risk of type 2 diabetes: a mendelian randomisation study. Lancet Diabetes Endocrinol.

[19] Hoell C, Wynn J, Rasmussen L (2020). Participant choices for return of genomic results in the eMERGE Network. Genetics Med.

[20] Lyall DM, Celis-Morales C, Lyall LM (2019). Assessing for interaction between APOE ε4, sex, and lifestyle on cognitive abilities. Neurology.

[21] Solomon A, Turunen H, Ngandu T (2018). Effect of the Apolipoprotein E genotype on cognitive change during a multidomain lifestyle intervention: a subgroup analysis of a randomized clinical trial. JAMA Neurol.

[22] Gelber RP, Petrovitch H, Masaki KH (2012). Lifestyle and the risk of dementia in Japanese-american men. J Am Geriatr Soc.

[23] Lourida I, Hannon E, Littlejohns TJ (2019). Association of lifestyle and genetic risk with incidence of dementia. JAMA.

[24] Licher S, Ahmad S, Karamujić-Čomić H (2019). Genetic predisposition, modifiable-risk-factor profile and long-term dementia risk in the general population. Nat Med.

[25] McCartney DL, Stevenson AJ, Walker RM (2018). Investigating the relationship between DNA methylation age acceleration and risk factors for Alzheimer's disease. Alzheimers Dement (Amst).

[26] Foley KE, Hewes AA, Garceau DT (2022). The APOEε3/ε4 Genotype Drives Distinct Gene Signatures in the Cortex of Young Mice. Front Aging Neurosci.

[27] Nott A, Schlachetzki JCM, Fixsen BR, Glass CK (2021). Nuclei isolation of multiple brain cell types for omics interrogation. Nat Protoc.

[28] Giacomelli E, Vahsen BF, Calder EL (2022). Human stem cell models of neurodegeneration: From basic science of amyotrophic lateral sclerosis to clinical translation. Cell Stem Cell.

[29] Grenier K, Kao J, Diamandis P (2020). Three-dimensional modeling of human neurodegeneration: brain organoids coming of age. Mol Psychiatry.

[30] Gerhartl A, Pracser N, Vladetic A, Hendrikx S, Friedl HP, Neuhaus W (2020). The pivotal role of micro-environmental cells in a human blood-brain barrier in vitro model of cerebral ischemia: functional and transcriptomic analysis. Fluids Barriers CNS.

[31] Mancuso R, Van Den Daele J, Fattorelli N (2019). Stem-cell-derived human microglia transplanted in mouse brain to study human disease. Nat Neurosci.

[32] Claes C, Danhash EP, Hasselmann J (2021). Plaque-associated human microglia accumulate lipid droplets in a chimeric model of Alzheimer’s disease. Mol Neurodegener.

[33] D’Angelo E, Jirsa V (2022). The quest for multiscale brain modeling. Trends Neurosci.

[34] Amunts K, Institute of Neurosciences and Medicine (INM-1) RCJ, Germany; C. & O. Vogt Institute for Brain Research, University Hospital Düsseldorf, Heinrich-Heine University Düsseldorf, Germany, ; Axer MB, Lise; Bjaalie, Jan; Brovelli, Andrea; Caspers, Svenja; Changeux, Jean-Pierre; Costantini, Irene; D'Angelo, Egidio; De Bonis, Giulia; Deco, Gustavo; DeFelipe, Javier; Destexhe, Alain; Dickscheid, Timo; Diesmann, Markus; Duqué, Julie; Düzel, Emrah; Eickhoff, Simon B.; Engel, Andreas K.; Evers, Kathinka; Fousek, Jan; Furber, Stephen; Goebel, Rainer; Güntürkün, Onur; De Kerchove d'Exaerde, Alban; Hellgren Kotaleski, Jeanette; Krsnik, Zeljka; Hilgetag, Claus C.; Hölter, Sabine M.; Ioannidis, Yannis; Jirsa, Viktor; Klijn, Wouter; Kämpfer, Julia; Lippert, Thomas; Maquet, Pierre; Marinazzo, Daniele; Meyer-Lindenberg, Andreas; Migliore, Michele; Morel, Yannick; Morin, Fabrice; Nagels, Guy; Oden, Lena; Panagiotaropoulos, Fanis; Paolucci, Pier Stanislao; Pennartz, Cyriel; Peeters, Liesbet M.; Petkoski, Spase; Petrovici, Mihai A.; Roelfsema, Pieter; Ris, Laurence; Ritter, Petra; Rotter, Stefan; Rowald, Andreas; Ruland, Sabine; Ryvlin, Philippe; Salles, Arleen; Sanchez-Vives, Maria V.; Schemmel, Johannes; Thirion, Betrand; Van Albada, Sacha Jennifer; Vanduffel, Wim; De Vos, Winnok. The coming decade of digital brain research - A vision for neuroscience at the intersection of technology and computing (Version 2.0). . Zenodo 2022.

[35] Hartl CL, Ramaswami G, Pembroke WG (2021). Coexpression network architecture reveals the brain-wide and multiregional basis of disease susceptibility. Nat Neurosci.

[36] Eraslan G, Drokhlyansky E, Anand S (2022). Single-nucleus cross-tissue molecular reference maps toward understanding disease gene function. Science.

[37] Rhrissorrakrai K, Belcastro V, Bilal E (2015). Understanding the limits of animal models as predictors of human biology: lessons learned from the sbv IMPROVER Species Translation Challenge. Bioinformatics.

[38] Perel P, Roberts I, Sena E (2007). Comparison of treatment effects between animal experiments and clinical trials: systematic review. BMJ (Clinical research ed).

[39] Ferrero J, Williams L, Stella H (2016). First-in-human, double-blind, placebo-controlled, single-dose escalation study of aducanumab (BIIB037) in mild-to-moderate Alzheimer's disease. Alzheimers Dement (N Y).

[40] Walsh S, Merrick R, Milne R, Brayne C (2021). Aducanumab for Alzheimer's disease?. BMJ.

[41] Budd Haeberlein S, Aisen PS, Barkhof F (2022). Two randomized phase 3 studies of aducanumab in early Alzheimer’s Disease. J Prev Alzheimer's Dis.

[42] The Lancet Digital H. Guiding better design and reporting of AI-intervention trials. Lancet Digit Health 2020;2:e493. 10.1016/S2589-7500(20)30223-5 33328046

[43] Harrer S, Shah P, Antony B, Hu J (2019). Artificial intelligence for clinical trial design. Trends Pharmacol Sci.

[44] Karki R, Kodamullil AT, Hoyt CT, Hofmann-Apitius M (2019). Quantifying mechanisms in neurodegenerative diseases (NDDs) using candidate mechanism perturbation amplitude (CMPA) algorithm. BMC Bioinformatics.

[45] Kodamullil AT, Younesi E, Naz M, Bagewadi S, Hofmann-Apitius M (2015). Computable cause-and-effect models of healthy and Alzheimer's disease states and their mechanistic differential analysis. Alzheimers Dement.

[46] Domingo-Fernández D, Kodamullil AT, Iyappan A (2017). Multimodal mechanistic signatures for neurodegenerative diseases (NeuroMMSig): a web server for mechanism enrichment. Bioinformatics.

[47] Godyń J, Jończyk J, Panek D, Malawska B (2016). Therapeutic strategies for Alzheimer's disease in clinical trials. Pharmacol Rep.

[48] Ryan J, Fransquet P, Wrigglesworth J, Lacaze P (2018). Phenotypic heterogeneity in dementia: a challenge for epidemiology and biomarker studies. Front Public Health.

[49] Friedman LG, McKeehan N, Hara Y (2021). Value-generating exploratory trials in neurodegenerative dementias. Neurology.

[50] Lantero Rodriguez J, Karikari TK, Suárez-Calvet M (2020). Plasma p-tau181 accurately predicts Alzheimer's disease pathology at least 8 years prior to post-mortem and improves the clinical characterisation of cognitive decline. Acta Neuropathol.

[51] Ashton NJ, Janelidze S, Al Khleifat A (2021). A multicentre validation study of the diagnostic value of plasma neurofilament light. Nat Commun.

[52] Rafii MS, Zaman S, Handen BL (2021). Integrating Biomarker Outcomes into Clinical Trials for Alzheimer's Disease in Down Syndrome. J Prev Alzheimers Dis.

[53] Jack CR, Bennett DA, Blennow K (2016). A/T/N: An unbiased descriptive classification scheme for Alzheimer disease biomarkers. Neurology.

[54] O'Connor A, Weston PSJ, Pavisic IM (2020). Quantitative detection and staging of presymptomatic cognitive decline in familial Alzheimer's disease: a retrospective cohort analysis. Alzheimers Res Ther.

[55] Weston PSJ, Nicholas JM, Henley SMD (2018). Accelerated long-term forgetting in presymptomatic autosomal dominant Alzheimer's disease: a cross-sectional study. Lancet neurol.

[56] Ayutyanont N, Langbaum JB, Hendrix SB (2014). The Alzheimer's prevention initiative composite cognitive test score: sample size estimates for the evaluation of preclinical Alzheimer's disease treatments in presenilin 1 E280A mutation carriers. J Clin Psychiatry.

[57] Bullain S, Doody R (2020). What works and what does not work in Alzheimer's disease? From interventions on risk factors to anti-amyloid trials. J Neurochem.

[58] Vogel JW, Young AL, Oxtoby NP (2021). Four distinct trajectories of tau deposition identified in Alzheimer's disease. Nat Med.

[59] Oxtoby NP, Alexander DC (2017). Imaging plus X: multimodal models of neurodegenerative disease. Curr Opin Neurol.

[60] Golriz K S, Robinson C, Birkenbihl C, Domingo-Fernández D, Hoyt CT, Hofmann-Apitius M. Challenges of Integrative Disease Modeling in Alzheimer's Disease. Front Mol Biosci 2020;6. 10.3389/fmolb.2019.00158 PMC6971060 31993440

[61] Young AL, Marinescu RV, Oxtoby NP (2018). Uncovering the heterogeneity and temporal complexity of neurodegenerative diseases with Subtype and Stage Inference. Nat Commun.

[62] Hascup ER, Hascup KN (2020). Toward refining Alzheimer's disease into overlapping subgroups. Alzheimers Dement (N Y).

[63] Oxtoby NP, Shand C, Cash DM, Alexander DC, Barkhof F (2022). Targeted Screening for Alzheimer's Disease Clinical Trials Using Data-Driven Disease Progression Models. Front Artif Intell.

[64] Brassington I (2017). The ethics of reporting all the results of clinical trials. Br Med Bull.

[65] Pérez-Mañá C, Llonch C, Farré M (2012). Transparency in clinical research: registration of clinical trials and publication of results. Med Clin (Barc).

[66] Tsai RM, Boxer AL (2016). Therapy and clinical trials in frontotemporal dementia: past, present, and future. J Neurochem.

[67] Marinescu R, Oxtoby N, Young A, et al. The Alzheimer's Disease Prediction Of Longitudinal Evolution (TADPOLE) Challenge: Results after 1 Year Follow-up2020.

[68] Goodday SM, Kormilitzin A, Vaci N (2020). Maximizing the use of social and behavioural information from secondary care mental health electronic health records. J Biomed Inform.

[69] Greicius MD, Srivastava G, Reiss AL, Menon V (2004). Default-mode network activity distinguishes Alzheimer's disease from healthy aging: evidence from functional MRI. Proc Natl Acad Sci U S A.

[70] Horvath A, Szucs A, Csukly G, Sakovics A, Stefanics G, Kamondi A (2018). EEG and ERP biomarkers of Alzheimer's disease: a critical review. Front Biosci (Landmark Ed).

[71] Stam CJ (2010). Use of magnetoencephalography (MEG) to study functional brain networks in neurodegenerative disorders. J Neurol Sci.

[72] Babiloni C, Blinowska K, Bonanni L (2020). What electrophysiology tells us about Alzheimer's disease: a window into the synchronization and connectivity of brain neurons. Neurobiol Aging.

[73] Chételat G, Arbizu J, Barthel H (2020). Amyloid-PET and 18F-FDG-PET in the diagnostic investigation of Alzheimer's disease and other dementias. Lancet Neurol.

[74] Klunk WE, Engler H, Nordberg A (2004). Imaging brain amyloid in Alzheimer's disease with Pittsburgh Compound-B. Ann Neurol.

[75] Lowe VJ, Curran G, Fang P (2016). An autoradiographic evaluation of AV-1451 Tau PET in dementia. Acta Neuropathol Commun.

[76] Jack CR, Bernstein MA, Fox NC (2008). The Alzheimer's Disease Neuroimaging Initiative (ADNI): MRI methods. J Magn Reson Imaging.

[77] Beekly DL, Ramos EM, Lee WW (2007). The National Alzheimer's Coordinating Center (NACC) database: the Uniform Data Set. Alzheimer Dis Assoc Disord.

[78] Marcus DS, Wang TH, Parker J, Csernansky JG, Morris JC, Buckner RL (2007). Open Access Series of Imaging Studies (OASIS): Cross-sectional MRI data in young, middle aged, nondemented, and demented older adults. J Cogn Neurosci.

[79] Rohrer JD, Nicholas JM, Cash DM (2015). Presymptomatic cognitive and neuroanatomical changes in genetic frontotemporal dementia in the Genetic Frontotemporal dementia Initiative ( GENFI ) study : a cross-sectional analysis. Lancet Neurol.

[80] Bauermeister S, Orton C, Thompson S (2020). The Dementias Platform UK (DPUK) Data Portal. Eur J Epidemiol.

[81] Li F, Liu M (2019). A hybrid convolutional and recurrent neural network for hippocampus analysis in Alzheimer's Disease. J Neurosci Methods.

[82] Morin A, Samper-Gonzalez J, Bertrand A (2020). Accuracy of MRI Classification Algorithms in a Tertiary Memory Center Clinical Routine Cohort. J Alzheimers Dis.

[83] Giorgio J, Landau S, Jagust W, Tino P, Kourtzi Z. Modelling prognostic trajectories of cognitive decline due to Alzheimer's disease. NeuroImage: Clinical 2020. 10.1016/j.nicl.2020.102199 PMC7044529 32106025

[84] Giorgio J, Jagust WJ, Baker S (2022). A robust and interpretable machine learning approach using multimodal biological data to predict future pathological tau accumulation. Nat Commun.

[85] Borchert R, Azevedo T, Badhwar A, et al. Artificial intelligence for diagnosis and prognosis in neuroimaging for dementia; a systematic review. medRxiv 2021:2021.2012.2012.21267677.

[86] Iturria-Medina Y, Sotero RC, Toussaint PJ (2016). Early role of vascular dysregulation on late-onset Alzheimer’s disease based on multifactorial data-driven analysis. Nat Commun.

[87] Vogel JW, Young AL, Oxtoby NP (2021). Four distinct trajectories of tau deposition identified in Alzheimer’s disease. Nat Med.

[88] Taheri Gorji H, Kaabouch N (2019). A Deep Learning approach for Diagnosis of Mild Cognitive Impairment Based on MRI Images. Brain Sci.

[89] Liu M, Li F, Yan H (2020). A multi-model deep convolutional neural network for automatic hippocampus segmentation and classification in Alzheimer’s disease. Neuroimage.

[90] Qiu S, Joshi PS, Miller MI (2020). Development and validation of an interpretable deep learning framework for Alzheimer’s disease classification. Brain.

[91] Varoquaux G, Cheplygina V. Machine learning for medical imaging: methodological failures and recommendations for the future. npj Digital Medicine 2022;5:48. 10.1038/s41746-022-00592-y PMC9005663 35413988

[92] Muehlematter UJ, Daniore P, Vokinger KN (2021). Approval of artificial intelligence and machine learning-based medical devices in the USA and Europe (2015–20): a comparative analysis. The Lancet Digital Health.

[93] Livingston G, Huntley J, Sommerlad A (2020). Dementia prevention, intervention, and care: 2020 report of the Lancet Commission. Lancet.

[94] Norton S, Matthews FE, Barnes DE, Yaffe K, Brayne C (2014). Potential for primary prevention of Alzheimer's disease: an analysis of population-based data. Lancet Neurol.

[95] Deckers K, van Boxtel MP, Schiepers OJ (2015). Target risk factors for dementia prevention: a systematic review and Delphi consensus study on the evidence from observational studies. Int J Geriatr Psychiatry.

[96] Bellou V, Belbasis L, Tzoulaki I, Middleton LT, Ioannidis JP, Evangelou E (2016). Systematic evaluation of the associations between environmental risk factors and dementia: An umbrella review of systematic reviews and meta-analyses. Alzheimers Dement.

[97] Anstey KJ, Ee N, Eramudugolla R, Jagger C, Peters R (2019). A Systematic review of meta-analyses that evaluate risk factors for dementia to evaluate the quantity, quality, and global representativeness of evidence. J Alzheimers Dis.

[98] Zhang Y, Xu W, Zhang W (2021). Modifiable Risk Factors for Incident Dementia and Cognitive Impairment: An Umbrella. Rev Evid.

[99] Parra KL, Alexander GE, Raichlen DA, Klimentidis YC, Furlong MA (2022). Exposure to air pollution and risk of incident dementia in the UK Biobank. Environ Res.

[100] Duchesne J, Gutierrez L-A, Carrière I (2022). Exposure to ambient air pollution and cognitive decline: Results of the prospective Three-City cohort study. Environ Int.

[101] Killin LOJ, Starr JM, Shiue IJ, Russ TC (2016). Environmental risk factors for dementia: a systematic review. BMC Geriatr.

[102] Kuźma E, Hannon E, Zhou A (2018). Which risk factors causally influence dementia? a systematic review of Mendelian randomization studies. J Alzheimers Dis.

[103] Nicholls HL, John CR, Watson DS, Munroe PB, Barnes MR, Cabrera CP (2020). Reaching the End-Game for GWAS: Machine Learning Approaches for the Prioritization of Complex Disease Loci. Front Genetics.

[104] Romagnoni A, Jégou S, Van Steen K (2019). Comparative performances of machine learning methods for classifying Crohn Disease patients using genome-wide genotyping data. Sci Rep.

[105] Chen L, Wang Y, Zhao F (2022). Exploiting deep transfer learning for the prediction of functional non-coding variants using genomic sequence. Bioinformatics.

[106] Prosperi M, Guo Y, Sperrin M (2020). Causal inference and counterfactual prediction in machine learning for actionable healthcare. Nature Machine Intelligence.

[107] Peters R, Booth A, Rockwood K, Peters J, D’Este C, Anstey KJ (2019). Combining modifiable risk factors and risk of dementia: a systematic review and meta-analysis. BMJ Open.

[108] Foote IF, Jacobs BM, Mathlin G (2022). The shared genetic architecture of modifiable risk for Alzheimer's disease: a genomic structural equation modelling study. Neurobiol Aging.

[109] Ma Y, Wolters FJ, Chibnik LB (2019). Variation in blood pressure and long-term risk of dementia: A population-based cohort study. PLoS Med.

[110] Perera G, Rijnbeek PR, Alexander M (2020). Vascular and metabolic risk factor differences prior to dementia diagnosis: a multidatabase case–control study using European electronic health records. BMJ Open.

[111] Lane CA, Barnes J, Nicholas JM (2019). Associations between blood pressure across adulthood and late-life brain structure and pathology in the neuroscience substudy of the 1946 British birth cohort (Insight 46): an epidemiological study. Lancet Neurol.

[112] Sproviero W, Winchester L, Newby D (2021). High blood pressure and risk of dementia: a two-sample Mendelian Randomization Study in the UK Biobank. Biol Psychiatry.

[113] Moore PJ, Lyons TJ, Gallacher J (2019). Using path signatures to predict a diagnosis of Alzheimer's disease. PLoS ONE.

[114] Sindi S, Calov E, Fokkens J, et al. The CAIDE Dementia Risk Score App: The development of an evidence-based mobile application to predict the risk of dementia. Alzheimer's dementia (Amsterdam, Netherlands). 2015;1:328–333. http://europepmc.org/abstract/MED/27239514;10.1016/j.dadm.2015.06.005;https://europepmc.org/articles/PMC4878198;https://europepmc.org/articles/PMC4878198?pdf=render. Accessed 2015/09//. 10.1016/j.dadm.2015.06.005 PMC4878198 27239514

[115] Kivipelto M, Ngandu T, Laatikainen T, Winblad B, Soininen H, Tuomilehto J (2006). Risk score for the prediction of dementia risk in 20 years among middle aged people: a longitudinal, population-based study. Lancet neurol.

[116] Liu R, Wei L, Zhang P (2021). A deep learning framework for drug repurposing via emulating clinical trials on real-world patient data. Nat Mach Intell.

[117] Fang J, Zhang P, Wang Q, et al. Network-based Translation of GWAS Findings to Pathobiology and Drug Repurposing for Alzheimer’s Disease. medRxiv 2020:2020.2001.2015.20017160.

[118] Xu J, Zhang P, Huang Y (2021). Multimodal single-cell/nucleus RNA sequencing data analysis uncovers molecular networks between disease-associated microglia and astrocytes with implications for drug repurposing in Alzheimer's disease. Genome Res.

[119] Yang F, Zhang Q, Ji X (2022). Machine Learning Applications in Drug Repurposing. Interdiscip Sci.

[120] Rodriguez S, Hug C, Todorov P, et al. Machine learning identifies candidates for drug repurposing in Alzheimer's disease. Nature commun. 2021;12:1033. http://europepmc.org/abstract/MED/33589615;10.1038/s41467-021-21330-0;https://europepmc.org/articles/PMC7884393;https://europepmc.org/articles/PMC7884393?pdf=render. Accessed Feb 2021. 10.1038/s41467-021-21330-0 PMC7884393 33589615

[121] Tsuji S, Hase T, Yachie-Kinoshita A (2021). Artificial intelligence-based computational framework for drug-target prioritization and inference of novel repositionable drugs for Alzheimer’s disease. Alzheimer's Res Ther.

[122] Tomlinson A, Furukawa TA, Efthimiou O (2020). Personalise antidepressant treatment for unipolar depression combining individual choices, risks and big data (PETRUSHKA): rationale and protocol. Evid Based Ment Health.

[123] Liu Q, Vaci N, Koychev I (2022). Personalised treatment for cognitive impairment in dementia: development and validation of an artificial intelligence model. BMC Med.

[124] Wang D, Liu S, Warrell J (2018). Comprehensive functional genomic resource and integrative model for the human brain. Science.

[125] Institute" UDR. A Multi-'omics Atlas Project - Alzheimer's Disease A UK DRI Director's Initiative. https://map-ad.org/.

[126] van der Worp HB, Howells DW, Sena ES (2010). Can animal models of disease reliably inform human studies?. PLoS Med.

[127] Ransohoff RM (2018). All (animal) models (of neurodegeneration) are wrongAre they also useful?. J Exp Med.

[128] Lotfollahi M, Naghipourfar M, Luecken MD (2022). Mapping single-cell data to reference atlases by transfer learning. Nat Biotechnol.

[129] Normand R, Du W, Briller M (2018). Found In Translation: a machine learning model for mouse-to-human inference. Nat Methods.

[130] You J, Zhang YR, Wang HF (2022). Development of a novel dementia risk prediction model in the general population: A large, longitudinal, population-based machine-learning study. EClinicalMedicine.

